# Efficacy of first-line doxorubicin and ifosfamide in myxoid liposarcoma

**DOI:** 10.1186/2045-3329-2-2

**Published:** 2012-01-24

**Authors:** Daniela Katz, Piyaporn Boonsirikamchai, Haeson Choi, Alexander J Lazar, Wei-Lein Wang, Lianchun Xiao, Min S Park, Vinod Ravi, Robert S Benjamin, Dejka M Araujo

**Affiliations:** 1 Department of Sarcoma Medical Oncology, The University of Texas MD Anderson Cancer Center, Houston, Texas, USA; 2 Department of Diagnostic Imaging, The University of Texas MD Anderson Cancer Center, Houston, Texas, USA; 3 Department of Pathology & Sarcoma Research Center, The University of Texas MD Anderson Cancer Center, Houston, Texas, USA; 4 Department of Biostatistics, The University of Texas MD Anderson Cancer Center, Houston, Texas, USA

**Keywords:** Choi criteria, doxorubicin, ifosfamide, myxoid liposarcoma

## Abstract

**Background:**

Myxoid liposarcoma (MLS) is a soft tissue sarcoma with adipocytic differentiation characterized by a unique chromosome rearrangement, t(12;16)(q13;p11). The exact efficacy of chemotherapy in MLS has not been clearly established.

**Patients and methods:**

We retrospectively analyzed the records of 37 histologically confirmed MLS patients who were treated at the University of Texas MD Anderson Cancer Center from January 2000 to December 2009 with doxorubicin 75-90 mg/m^2 ^over 72 hours combined with ifosfamide 10 gm/m^2 ^in the first-line setting. Response was assessed using RECIST and Choi criteria. The Kaplan-Meier method and log-rank test was used to estimate clinical outcomes.

**Results:**

The median follow-up period was 50.1 months. The overall response rates were 43.2% using RECIST and 86.5% using the Choi criteria. The 5-year disease-free survival rate was 90% for patients with resectable tumors. Median time to progression and overall survival time for the advanced-disease group were 23 and 31.1 months, respectively.

**Conclusion:**

Our study demonstrates that doxorubicin-ifosfamide combination therapy has a role in the treatment of MLS. The Choi criteria may be more sensitive in evaluating response to chemotherapy in MLS.

## Introduction

Liposarcoma is the name given to a group of soft tissue sarcomas (STSs) with adipocytic differentiation. As a group, the liposarcomas are the second most common STS in adults. Approximately half of these tumors are further sub-classified as myxoid/round cell liposarcomas (MLSs) based on a multinodular gelatinous appearance and a unique chromosome rearrangement, t(12;16)(q13;p11), involving the *DDIT3 *and *FUS *genes, respectively. This chromosome rearrangement or a rare variant in which *FUS *is substituted by *EWSR1 *(22q12) is found in virtually all MLS cases and supports the diagnosis [1]. MLS exhibits distinct clinical features, such as a propensity to develop in the lower extremity, particularly the medial thigh or the popliteal area, and only very rarely in the retroperitoneum as a primary site [2]. Furthermore, compared with other STSs, it has a strong predisposition to metastasize to nonpulmonary sites such as the intraperitoneum, the retroperitoneum, or the paraspinal fat [2-4]. Staging and grading of these tumors are no different from those of other STSs, with the extent of the round-cell change/hypercellularity component in the tumor reflecting aggressiveness [5].

As with other STSs, the cornerstone of successful treatment for localized MLS disease is surgical excision. Radiation is part of the multidisciplinary approach commonly applied to tumors larger than 5 cm [6]. Chemotherapy is added for tumors larger than 10 cm and may be used for selected tumors in the 5- to 10-cm range, and it is the mainstay treatment of metastatic disease. Doxorubicin and ifosfamide in combination (AI) are often used as a first-line regimen in previously untreated patients with STS, with response rates of 10-66% [7-9]. Earlier studies have shown that STS response to AI follows a positive dose-response curve, with doxorubicin given at doses of 75 mg/m^2 ^and ifosfamide at 10 gm/m^2 ^providing the best efficacy-to-toxicity ratio [10-12]. As a result, this regimen was adopted a decade ago as the standard of care for first-line STS patients 65 years and younger at The University of Texas MD Anderson Cancer Center. Experience acquired at our institution with this dose-intensive regimen has been particularly promising for MLS tumors, which have had a higher response rate than have other STSs. However, the sarcoma literature is vague on the response rates of MLS to AI, mainly because studies often report results with all liposarcoma subtypes pooled [11,13], a disadvantage for MLSs, which appear to be more sensitive than other liposarcomas to chemotherapy. In support of the chemotherapy responsiveness of MLS, the few studies that have addressed the response of MLS alone to different chemotherapy regimens, such as a doxorubicin-based regimen or single-agent trabectedin (Yondelis), demonstrate an overall response rate of 45-50% [14-16].

The fact that studies have not specifically addressed the outcome of MLS patients treated with doxorubicin 75 mg/m^2 ^and ifosfamide 10 gm/m^2 ^dosing regimen prompted us to retrospectively evaluate our institutional experience with first-line AI for chemotherapy-naïve patients with localized or advanced MLS. Response was assessed using both Response Evaluation Criteria in Solid Tumors (RECIST) [17] and Choi criteria [18].

## Patients and Methods

### Patients

Patients were identified by searching the MD Anderson computerized database for a diagnosis of MLS between January 2000 and December 2009. Review of data began after approval from our institutional review board. We identified 37 chemotherapy-naïve patients who had either localized (resectable) or advanced (metastatic or nonresectable) measurable MLS. These patients were then treated with up to eight cycles of AI. Demographic data, tumor characteristics, chemotherapy dosage and schedule, radiotherapy administration, surgical margin status, and patient outcome were extracted from clinical records. Toxicity data (dose reduction, discontinuation of chemotherapy, and death during treatment) were gathered. All imaging studies, including computed tomography (CT) and magnetic resonance imaging (MRI) scans, were reviewed by a single MD Anderson radiologist (P.B.). Tissue diagnosis of MLS was confirmed in each case independently by two MD Anderson sarcoma pathologists (A.J.L., W.-L.W.).

### Treatment

The 37 patients in this retrospective review were treated according to the Department of Sarcoma Medical Oncology's standard approach for high-grade STSs larger than 5 cm, which is a combination of chemotherapy, radiotherapy, and definitive surgery when feasible. These patients received doxorubicin (75-90 mg/m^2 ^as a continuous infusion over 72 hours every 3 weeks) combined with ifosfamide (10 gm/m^2 ^given in divided doses of 2.5 gm/m^2 ^infused over 3 hours daily for 4 days, every 3 weeks). Radiotherapy was administered either before or after surgery following the recommendations of our institution's sarcoma multidisciplinary team comprised of sarcoma surgeons, radiotherapists, radiologists, and sarcoma medical oncologists. Baseline and follow-up imaging were performed with CT and/or MRI every two cycles to assess response.

### Endpoints

The primary study endpoint was to assess objective response to treatment with first-line AI. RECIST [17] and Choi criteria [18] were used for evaluation of response. For tumors that were imaged with MRI, the density response aspect of the Choi criteria were not evaluable; only the size aspect was used for response assessment.

### Statistical analysis

Fisher's exact test was used to determine the association of response and categorical variables, as well as to compare some patient characteristics between the resectable- and advanced-disease groups. The Wilcoxon rank sum test was used to compare each of the continuous variables between patients with PR and patients with stable disease and between resectable- and advanced-disease patients. The Kaplan-Meier method and log-rank test were used to analyze time-to-event variables, including overall survival and disease-free survival.

## Results

### Patient characteristics

Corresponding clinical data and imaging were reviewed for 37 patients. Table 1 summarizes the clinical data. All patients were chemotherapy-naïve and received AI as part of their initial treatment. Twenty-six patients received neoadjuvant AI for localized tumors, and 11 patients received first-line AI for advanced disease (four presented with metastatic disease, and seven had distant relapse). The vast majority of primary tumors were found in the lower extremity, while advanced disease involved bones, the abdomen, and the upper trunk (Table 1).

**Table 1:** Patient characteristics.

*Characteristic*	*Resectable tumor No. (%)*	*Advanced disease No. (%)*	*Total No. (%)*	*P value*
**Total no. of patients**	26	11	37	

**Sex**				0.48

**Male**	15 (57.7)	3 (27.3)	18 (48.6)	

**Female**	11 (42.3)	8 (72.7)	19 (51.4)	

**Race**				0.35

**White**	15 (57.7)	10 (90.9)	25 (67.6)	

**Hispanic**	6 (23.0)	1 (9.1)	7 (18.9)	

**African American**	4 (15.4)	0 (0)	4 (10.8)	

**Asian**	1 (3.8)	0 (0)	1 (2.7)	

**Age in years at the time of treatment, mean ± SD (range)**	42.3 ± 12.8 (14-72)	43.8 ± 6.4 (37-55)	42.7 ± 11.4 (14-72)	0.87

**Tumor size in cm, median (range)**	14.9 (6-23.3)			

**Disease site**				

**Lower extremity**	23 (88.5)			

**Neck**	1 (3.8)			

**Retroperitoneum**	1 (3.8)			

**Perineum**	1 (3.8)			

**Number of disease sites**				

**Single site (abdominal cavity, bone^a^, heart, mediastinum)**		5 (45.5)		

**2 sites**^**b**^		5 (45.5)		

**3 sites**^**c**^		1 (9.1)		

**Tumor side**				

**Left**	15 (57.7)			

**Right**	11 (42.3)			

**Hypercellular round cell tumor**	1 (3.8)	1 (9.1)	2 (5.4)	0.82

**Chemotherapy**				

**Median no. of chemotherapy cycles, median (range)**	6 (4-6)	6 (5-8)	6 (4-8)	0.02

**Intensified doxorubicin dose (90 mg/m^2^)**	8 (30.7)	3 (27.2)	11 (33.3)	1.00

**Surgery (R0 intent)**	26 (100)			

**Free margins**	21 (80.8)			

**Positive margins (R1)**	5 (19.2)			

**Radiotherapy**				

**Before surgery**	21 (80.8)			

**After surgery**	5 (19.2)			

^a^All bony involvement is reported as a single site (involved bones: sternum and spine, orbit and rib).

^b^Involed sites: lung and sacrum, retroperitoneum and chest wall, sacrum/T5 and peritoneum, sacrum and buttock, mediastinum and tibia (local recurrence).

^c^Involved sites: Pelvis, lung and abdominal cavity implants.

### Treatment adherence and safety

All patients treated with neoadjuvant AI for a resectable tumor received between four and six cycles, with 17 of 26 patients (65%) receiving six cycles and only two patients receiving four cycles (Table 1). No patient demonstrated progressive disease. The reasons for administering fewer than six cycles were patient's preference (three patients), maximum tumor response (three patients), secondary tumor resistance (one patient), and myelosuppression (two patients). For patients with advanced disease, between five and eight cycles of AI were administered. For these patients, AI chemotherapy was discontinued because of maximum response (seven patients), progression after the fifth cycle (one patient), and patient preference to discontinue treatment after the sixth cycle (one patient). Doxorubicin at an intensified dose of 90 mg/m^2 ^was administered to 11 patients (eight with resectable disease and three with advanced disease). In general, patients tolerated the AI regimen well. Only two patients had prolonged myelosuppression, which led to the discontinuation of chemotherapy after the fifth cycle. In one patient, the doxorubicin delivery duration was decreased to 48 hours because of mucositis. No toxicity-related deaths occurred during treatment.

### Response and outcome

**Figure 1:**
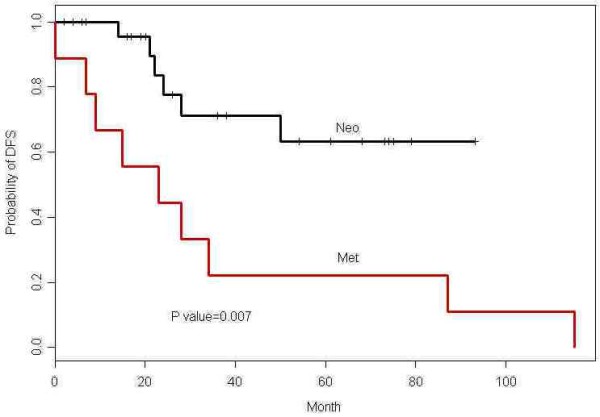
**Disease-free survival in patients with resectable tumors (Neo) and in patients with advanced disease (Met)**.

**Figure 2:**
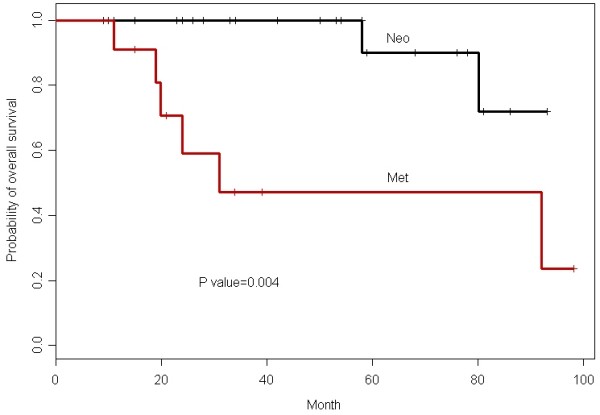
**Overall survival in patients with resectable tumors (Neo) and in patients with advanced disease (Met)**.

Table 2 summarizes responses determined using the RECIST and Choi criteria. Patients with resectable disease and patients with advanced disease did not differ significantly in response rates to AI (RECIST, p = 0.48; Choi criteria, p = 1.00). The PR rate for the entire cohort was 43% (n = 16) by RECIST and 87% (n = 32) by Choi criteria. The maximum tumor diameter decreased by an average of 28% (median 26%, range 5-57%.), and the mean time to maximum response was 4.4 months (median 4 months, range 2-10 months). No complete responses or disease progressions during preoperative treatment were recorded, and all patients with resectable disease underwent surgery with an R0 (curative) intent. Median follow-up for the entire group was 50.1 months (95% CI, 34-76.1 months). Six patients who received preoperative chemotherapy (two with positive margins) developed distant metastases diagnosed at a median of 23 months (range, 14-50 months) following surgery; two of these six patients died. The disease-free survival rate at 5 years for patients with resectable tumors was 90% (95% CI, 73.2-100%) (Figure 1). For the advanced-disease group, median time to progression (TTP) was 23 months (95% CI, 9.01-NA), and median overall survival was 31.1 months (95% CI, 20-NA) (Figure 2).

**Table 2:** Efficacy of AI and clinical outcome

	*Resectable tumor No. (%)*	*Advanced disease No. (%)*	*Total No. (%)*	*P value*
**Response by RECIST**				0.48

**PR**	10 (38.5)	6 (54.5)	16 (43.2%)	

**SD**	16 (61.5)	5 (45.5)	21 (56.8%)	

**Response by Choi criteria**				1.00

**PR**	22 (84.6)	10 (91)	32 (86.5%)	

**SD**	4 (15.4)	1 (9)	5 (13.5%)	

**Maximum % decrease in tumor size in cm, median (range)**	26.3 (6.2-56.1)	29.9 (4.5-56.8)	27.6% (4.5-56.8)	0.5

**Time to maximal response in months, median (range)**	4 (2-10)	4 (3-7)	4 (1.9-10.1)	0.44

**5-years DFS**	90%			(95% CI, 73.2 to 100)

**TTP in months, median**		23		(95% CI, 9.01 to NA)

**OS in months, median**		31.1		(95% CI, 20 to NA)

Abbreviations: PR partial response, SD stable disease, DFS disease-free survival,

When Fisher's exact test was applied, no significant association was detected for response evaluated by either RECIST or Choi criteria with age, sex, tumor size, resectability, primary or advanced disease, round-cell change/hypercellularity, doxorubicin dose (75 or 90 mg/m^2^), or number of cycles of chemotherapy. Of course, the numbers in any of these subgroups were very small.

## Discussion

Inherently, MLSs are very chemotherapy-responsive tumors, and this modern series confirms that finding. The dose-intensive regimen of doxorubicin (75-90 mg/m^2^) and ifosfamide (10 gm/m^2^) in this study attained a PR rate of 43.2% by RECIST and 86.5% by Choi criteria for the entire cohort. No patient had progressive disease as the initial response to therapy. The efficacy of this regimen was not statistically different between patients with resectable disease and patients with advanced disease. We were encouraged by the high response rate, accompanied by a lack of disease progression, observed during the first four to six cycles, which highlights the utility of chemotherapy in the preoperative setting, in which chemotherapy is likely to shrink the tumor or kill tumor cells.

The high efficacy of dose-intensive AI in MLS appears to be not a feature unique to this regimen but rather a feature of the chemotherapy sensitivity of MLS. Patel et al. [16] reported a response rate of 44% (by RECIST) in 18 patients treated with first-line doxorubicin (60-90 mg/m^2 ^as a 48- to 96-hour continuous infusion) and dacarbazine (900-1000 mg/m^2^) with or without cyclophosphamide (600-750 mg/m^2^). Grosso et al. [15] reported a high response rate of 51% per RECIST in a retrospective study of patients treated with trabectedin and noted that a substantial number of these patients (74%) demonstrated what their group termed tissue response prior to tumor shrinkage. These tissue responses would all qualify as Choi responses. Similar response rates were recently reported in a prospective study by Gronchi et al. [14]. In that phase II study, 13 patients were treated preoperatively with first-line trabectedin (1.5 mg/m^2 ^every 3 weeks) and had a 46% response rate by RECIST with no progressions noticed during the neoadjuvant period.

In addition to being chemotherapy sensitive, MLSs are also quite radiotherapy sensitive, as demonstrated by Guadagnolo et al. [19]. In their study, radiotherapy given before or after surgery to 128 MLS patients resulted in a 97% 5-year local control rate, accompanied by an 81% 5-year disease-free survival rate and 87% overall survival rate. The high efficacy of chemotherapy and radiotherapy in MLS suggests they should play a role whenever surgical morbidity or organ functionality preservation issues arise in the neoadjuvant setting.

Based on the definitions of the Choi criteria, it is not a surprise that their use results in a significantly higher response rate than is seen with RECIST. Choi criteria set the bar for PR lower, identifying PR as a 10% decrease in tumor size, compared with 30% using RECIST. Bearing this difference in mind from a clinical perspective, Choi criteria better mirror day-to-day practice in the clinic, where decisions about chemotherapy continuation often need to be made based on subtle changes in tumor size. However, the major caveats for the Choi criteria seem to be twofold: First, although the method has been validated in assessing responses in gastrointestinal stromal tumors treated with imatinib mesylate [17], it is not yet widely accepted nor utilized for monitoring responses to chemotherapy in other tumor types. However, we believe that monitoring and reporting responses by both RECIST and Choi criteria, as was done in this study, is more informative than RECIST alone because the Choi criteria capture more subtle changes in tumor response and provide a more accurate assessment for oncologists and their patients regarding the probability of benefit from chemotherapy.

The second caveat for the Choi criteria is that for determining density, a CT scan with contrast is required. This aspect of the Choi criteria is less amenable to primary MLS since the majority of tumors originate in an extremity, where MRI is often the preferred imaging modality. Nevertheless, since changes in tumor size may be insufficient to represent actual tumor response, Stacchiotti et al. [20] suggested MRI with contrast material enhancement to complement tumor size, thus making Choi criteria a predictive tool of pathologic response in high-grade STS. Interestingly, decreased contrast enhancement, either on CT or MRI, following chemotherapy appears to be a predictor of pathological response.

The current study was limited by its retrospective nature that precluded strict follow-up as well as toxicity monitoring. Nevertheless, from our long-standing experience with this dose-intensive regimen, its toxicity is manageable and involves mostly myelosuppression; only two patients out of 37 in our study had to discontinue treatment, and in both cases discontinuation was secondary to prolonged myelosuppression. Severe potential forms of toxicity related to this regimen, such as cardiotoxicity, nephrotoxicity, and neurotoxocity, are relatively rare in our sarcoma center due to stringent follow-up and the employment of a series of preventive measures, such as the use of continuous-infusion doxorubicin treatment over 72-96 hours or bolus administration with dexrazoxane (Zinecard) in lieu of bolus doxorubicin alone; generous hydration combined with forced diuresis when needed; monitoring of fluid status and creatinine levels on a daily basis; and supplementing with bicarbonate and electrolytes such as potassium, magnesium, and phosphorus. Finally, setting a serum albumin lower limit of 3 gm/dl for initiating treatment with ifosfamide and supplementing with intravenous albumin significantly decrease neurotoxicity. In addition to the regimen-related preventive measures above, routine supportive care, including antibiotics, antiemetics, and growth factors, is also provided to all patients.

This study was also limited by the small number of patients, which precluded further analysis for any association between response and other variables such as age, sex, round-cell change/hypercellularity, doxorubicin dose (75 or 90 mg/m^2^), or number of cycles.

To our knowledge, this is the first report describing response to first-line AI in MLS. By reporting responses by both RECIST and Choi criteria, we were able to compare our findings with other studies that used the more conventional RECIST alone. We conclude that MLS is a chemotherapy-responsive tumor and that AI dose-intensive chemotherapy is an effective and safe approach for MLS. We advocate the use of more sensitive criteria such as the Choi criteria in evaluating response to chemotherapy.

## Competing interests

The authors declare that they have no competing interests.

## Authors' contributions

DK extracted patient data from clinical records and drafted the manuscript. PB reviewed all imaging studies. HC reviewed some of the imaging studies. AL confirmed tissue diagnosis of all of the cases. W-LL confirmed tissue diagnosis of all of the cases. LX carried out the statistical analysis. MP helped edit the manuscript. VR set up the initial database and identified the patient cases from our tumor registry. RB conceived of the study. DA helped draft and edit the manuscript and coordinated the manuscript. All authors read and approved the final manuscript.
